# Learned feature regularities enable suppression of spatially overlapping stimuli

**DOI:** 10.3758/s13414-022-02612-1

**Published:** 2022-11-23

**Authors:** Daniel D. Thayer, Maggie Miller, Barry Giesbrecht, Thomas C. Sprague

**Affiliations:** Department of Psychological and Brain Sciences, University of California, Santa Barbara, Santa Barbara, CA 93106-9660 USA

**Keywords:** Attention, Statistical learning, Distractor suppression

## Abstract

Contemporary theories of attentional control state that information can be prioritized based on selection history. Even though theories agree that selection history can impact representations of spatial location, which in turn helps guide attention, there remains disagreement on whether nonspatial features (e.g., color) are modulated in a similar way. While previous work has demonstrated color suppression using visual search tasks, it is possible that the location corresponding to the distractor was suppressed, consistent with a spatial mechanism of suppression. Here, we sought to rule out this possibility by testing whether similar suppression of a learned distractor color can occur for spatially overlapping visual stimuli. On a given trial, two spatially superimposed stimuli (line arrays) were tilted either left or right of vertical and presented in one of four distinct colors. Subjects performed a speeded report of the orientation of the “target” array with the most lines. Critically, the distractor array was regularly one color, and this high-probability color was never the color of the target array, which encouraged learned suppression. In two experiments, responses to the target array were fastest when the distractor array was in the high-probability color, suggesting participants suppressed the distractor color. Additionally, when regularities were removed, the high-probability distractor color continued to benefit speeded target identification for individual subjects (E1) but slowed target identification (E2) when presented in the target array. Together, these results indicate that learned suppression of feature-based regularities modulates target detection performance independent of spatial location and persists over time.

**Supplementary Information:**

The online version contains supplementary material available at 10.3758/s13414-022-02612-1.

## Introduction

The visual system is constantly bombarded with information, of which only a small portion can be attended. When searching the kitchen for ingredients to make pizza, features and locations in the kitchen that are aligned with the goal of making pizza get prioritized. For example, one may prioritize search for the red tomato sauce in the cabinet. Search for pizza-related items can be disrupted, such as when there is a salient, unexpected, and abrupt appearance of a roommate in the kitchen. The processing of other salient items in the scene, such as the bright green parsley growing in the window, are not disruptive, but instead might be suppressed due to their regular presence in the kitchen. The ability to prioritize specific information based on one’s goals, the automatic capture from abrupt onset salient stimuli, and the learned suppression of regularly presented items all interact to produce the phenomenon of attentional control (Awh et al., 2012; Luck et al., 2020).

The interplay among these signals has been characterized within the priority map framework (Itti & Koch, 2001; Koch & Ullman, 1985; Treisman & Gelade, 1980; Wolfe, 1994; Zelinsky & Bisley, 2015), where a priority map reflects the importance of specific locations within the visual field. To compute a feature-agnostic priority map, individual maps of specific feature dimensions (e.g., color or orientation), which contain information corresponding to locations that are important based on being physically salient as well as based on their relevance for ongoing goals, are summed. Bottom-up and top-down inputs have been well established to drive attentional selection through behavioral (Bundesen, 1990; Duncan & Humphreys, 1989; Olivers et al., 2006) and neural (Fecteau & Munoz, 2006; Gottlieb et al., 1998; Serences & Yantis, 2006) studies and can modulate priority at the level of individual feature maps (McMains et al., 2007; Runeson et al., 2013; Saenz et al., 2002; Serences & Boynton, 2007) or an integrated priority map (Bisley & Goldberg, 2003, 2006; Bogler et al., 2011, 2013).

The contribution of a third category, selection history, has been proposed due to results that do not adhere to the canonical top-down/bottom-up dichotomy (Awh et al., 2012; Shomstein et al., 2022). Selection history is distinct from top-down attention, as the influence of previous deployments of attention modulate priority without the explicit awareness of an individual and can even interfere with ongoing goals (Hickey et al., 2010). Additionally, selection history is distinct from bottom-up salience because selection history clearly cannot influence the physical properties of stimuli which render them salient.

One primary means by which selection history influences the allocation of attention is by deprioritizing regularly presented distractors (Gaspelin et al., 2019; Stilwell et al., 2019; Wang & Theeuwes, 2018). Such distractor suppression is often studied using the additional singleton paradigm (Theeuwes, 1991, 1992). Briefly, this task commonly involves searching for a target shape among various distractor shapes, such as a target diamond among circle distractors. On some trials, a critical distractor appears that is presented in a distinct color from the rest of the display (e.g., red distractor among green items). When present, this distractor tends to slow response times (RTs), which is due to attention being directed to the location of the distractor based on its salience (Jonides & Yantis, 1988; Theeuwes et al., 2003). However, when the critical distractor is regularly presented at a specific position within the search array, capture effects are diminished (Stilwell et al., 2019), or even completely abolished such that performance is the same as distractor-absent trials (Wang & Theeuwes, 2018). This modulation occurs without explicit knowledge of the location regularities (Gao & Theeuwes, 2022), indicating a process distinct from top-down influences.

Suppression is thought to occur via two mechanisms: proactive inhibition and reactive rejection (Geng, 2014). Proactive inhibition deprioritizes information prior to the onset of a visual display. For instance, fewer saccades are directed towards the location where a singleton was usually presented than any other location (Gaspelin et al., 2019; Stilwell & Vecera, 2022), consistent with the possibility that the learned location was suppressed prior to display onset. Reactive mechanisms involve the rapid disengagement from distracting information after covert or overt attention has already been captured (Theeuwes, 2010). They are thought to act primarily within a spatial context, as evidence shows suppression restricted to a specific location (Theeuwes et al., 2003). Thus, mechanisms of suppression likely act on a feature-agnostic priority map, and not necessarily at the level of individual feature dimension maps (Luck et al., 2020). This raises the question: To what extent do nonspatial stimulus features (e.g., color hue, shape) contribute to distractor suppression?

The additional singleton paradigm lends itself to investigating the learned suppression of features such as color (Failing et al., 2019; Stilwell & Gaspelin, 2021; Vatterott & Vecera, 2012). For example, Stilwell et al. (2019) reported that when the location of a singleton is completely randomized, but presented in one high-probability color, RTs were faster than when the singleton was a low-probability color. This is consistent with participants suppressing specific color values when beneficial for task performance. However, an important aspect of the visual search tasks used in previous research is that each item in the display has a distinct spatial position, which inserts ambiguities on whether feature control mechanisms were implemented independent of space; it could be the case that only after a feature singleton captures attention, then reactive mechanisms suppress the *location* corresponding to the salient singleton (Luck et al., 2020; Moher & Egeth, 2012; Theeuwes et al., 2003). One way to disentangle the influences of features and space is to demonstrate feature-specific deprioritization *independent* of location.

**Fig. 1:**
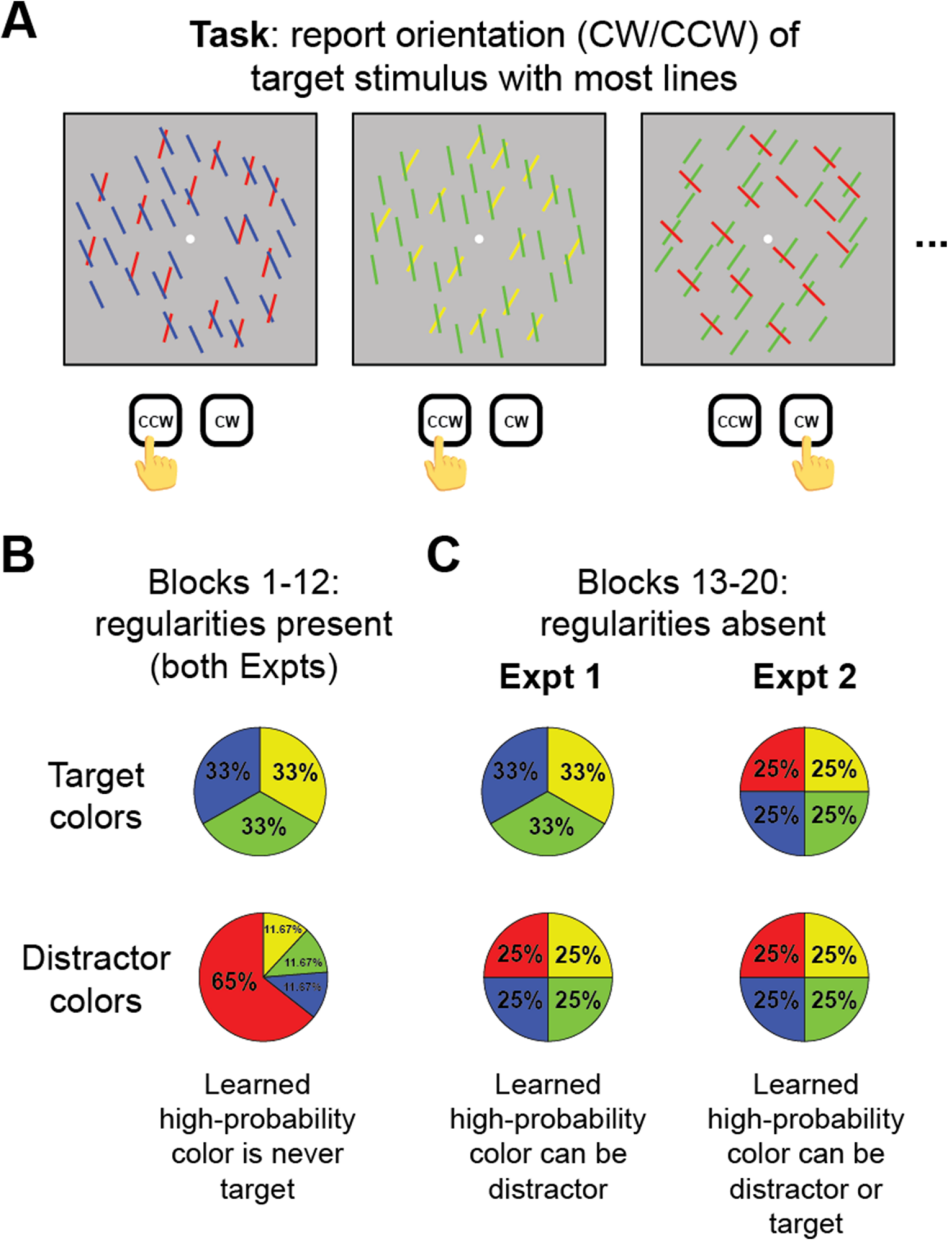
Discrimination task. **A** On each trial, participants were shown two oriented line arrays, each presented in one of four different equiluminant colors. One array was tilted clockwise from vertical, and the other was counterclockwise from vertical. Participants determined which array had the most lines, and then reported the orientation of that array with a button press. **B** Color regularities were present during the first 12 blocks of both experiments, such that the array with fewer lines was usually presented in the high-probability color (65% of trials). The target array was never presented in the high-probability color when regularities were present. **C** Regularities were removed in the last eight blocks of both experiments, meaning that the array with the fewest lines had an equal chance of being any of the four possible colors (25%). In Experiment 1, the array with the most lines was still never presented in the previously high-probability distractor color. Experiment 2 allowed both the target and distractor line arrays to be shown in any of the four colors with equal probability. The arrays were never presented in the same color on a given trial. Images here are illustrative cartoons; actual colors were equiluminant and line density/orientation are described in detail in Methods. (Color figure online)

A common procedure to minimize the impact of space is to use overlapping stimuli (Duncan, 1984; Giesbrecht et al., 2003; Liu et al., 2003; O’Craven et al., 1999; Saenz et al., 2002; Yantis & Serences, 2003). This way, spatial location is shared among stimuli, which isolates feature-specific mechanisms and minimizes the ability of a spatially driven mechanism to selectively suppress one, but not another, stimulus. We adopted this strategy in the current study by having participants perform an orientation discrimination task on two spatially overlapping colored line arrays. In this task (Fig. 1A), participants identified which of two arrays had more lines, then determined the orientation of the higher-density (“target”) line array. Critically, the low-density (“distractor”) array was typically presented in one color (Fig. 1B). If feature control mechanisms can specifically suppress the representation of a stimulus without necessarily suppressing all stimuli at a given location, then we expected behavioral performance to be faster when the distractor array was presented in the high-probability color. However, if reactive mechanisms are suppressing the location corresponding to the distractor array, then the target array would also be suppressed due to their spatial overlap. If this latter account is true, then we would expect to see no difference in behavioral performance whenever the distractor array was shown in the high-probability color or any of the low-probability colors.

Additionally, it is imperative to pinpoint the duration of suppression effects, as it is informative about the mechanism of prioritization (Wöstmann et al., 2021). Studies have shown that intertrial priming, or the influence of the previous trial on current trial performance (Maljkovic & Nakayama, 1994), and statistical learning, or the extraction of long-term display regularities to adjust future performance (Jiang, 2018; Jiang et al., 2013; Vatterott & Vecera, 2012), both influence distractor suppression.

To isolate the duration of feature suppression, we included several blocks in which color regularities were removed from the display. In Experiment 1, during these regularity-absent blocks, the distractor array had an equal chance of being shown in any of the possible colors (Fig. 1C). Whereas, in Experiment 2, both the target and distractor array had an equal chance of being presented in the previously high-probability color (Fig. 1C). These regularity-absent blocks allowed us to determine the specific mode of suppression by evaluating whether effects persisted after learning blocks, consistent with statistical learning, or whether they were primarily driven by intertrial priming within the learning phase itself.

In both experiments, we found robust suppression of the high-probability distractor color when regularities were present. Furthermore, subjects who showed the suppression effect when regularities were present continued to suppress the high-probability color when regularities were absent. In Experiment 2, we found additional evidence for long-term distractor suppression, as RTs were slower when the target array was presented in the previously learned high-probability distractor color. Overall, our results demonstrate that learned distractor colors can be suppressed independent of a spatial suppression mechanism, and that this suppression is supported by statistical learning of distractor feature values.

## Experiment 1

The goal of Experiment 1 was to determine whether feature-specific suppression occurs when stimuli are spatially overlapping. If so, this would suggest that feature control mechanisms can be independent of spatial control operations. We also sought to test whether suppression was transient, consistent with intertrial priming, or whether suppression persisted over longer periods of time, consistent with statistical learning.

### Method

#### Participants

The study protocol was approved by the UCSB institutional review board. Twenty-four participants (16 female, mean age = 18.5 years) were recruited from the University of California, Santa Barbara (UCSB) subject pool. All participants reported normal or corrected-to-normal vision and either received course credit or $10/hr upon completing the experimental session. Participants gave written consent prior to participating in the study. Previous work investigating color suppression using a visual search task (Stilwell et al., 2019) reported an effect size of $$ {\eta}_p^2 $$ = 0.68 and a power analysis using this effect size indicated that four subjects were needed to obtain 80% power. Since our study used a different task, we collected data from 24 participants to ensure enough statistical power to detect effects in our experiments.

#### Apparatus and stimuli

Participants viewed stimuli in a darkened room on a 25-in LED-backlit LCD screen with a resolution of 2,560 × 1,440 pixels. They were seated approximately 60 cm away from the screen. Stimuli were presented using MATLAB and Psychtoolbox (Brainard, 1997).

A white (80.1 cd/m^2^) dot centered at fixation was presented at the start of each block with a radius of 0.15° visual angle against a gray (49.4 cd/m^2^) background (Fig. 1). The fixation stimulus was visible throughout the whole block. On each trial, two oriented line arrays were presented. All lines in one array were oriented 45° clockwise of vertical, while the lines of the other array were oriented 45° counterclockwise. The orientation of the line arrays was randomized on each trial. Jitter was independently added to the orientation of both arrays randomly selected from 0.3°–1.2° orientation. Both arrays were presented within an imaginary circle with a radius of 10.5° visual angle. One array always contained 60 ± 20 (randomly selected on each trial) more lines than the other array. The array with more lines was the “target,” while the other array was the “distractor.” The number of lines in the target array had a range of 150 to 170 lines, while the distractor array could contain 90–110 lines. Individual lines had a length of 1.5° visual angle and a width of 0.05° visual angle. The color of either array was selected from the following four isoluminant colors in CIE color space: green (40.7 cd/m^2^, *x* = 0.243, *y* = 0.397), red (40.6 cd/m^2^, *x* = 0.421, *y* = 0.285), blue (40.3 cd/m^2^, *x* = 0.182, *y* = 0.175), and yellow (40.3 cd/m^2^, *x* = 0.450, *y* = 0.481). The target array was always a different color from the distractor array. Feedback text at the end of each block was presented in gray Arial font (RGB: 100, 100, 100). Participants reported whether the target array was oriented clockwise or counterclockwise from vertical with a left or right button press using a USB response pad.

#### Design and procedure

The fixation dot was presented at the start of the experiment and was visible throughout the whole block of 60 trials. At the start of each trial, the fixation dot was presented alone for 1,000 ms. Participants were instructed to attend and fixate the central dot until stimulus array onset. Next, the target and distractor line arrays were presented for up to 3,000 ms or until response. Participants determined whether there were more lines tilted to the counterclockwise or clockwise of vertical and reported the corresponding orientation with a left/right button press. They were encouraged to respond as fast as possible while still being accurate. At the start of the experiment, a random color was selected to be the prevalent distractor color for each subject (selected from red, green, blue, and yellow). During the first 12 blocks of the experiment, on 65% of trials, the distractor array was presented in the selected high-probability color. For the remaining 35% of trials, the distractor array was equally presented in one of the other three low-probability colors (11.67% of trials for each remaining color). The target array was never presented in the high-probability distractor color. The color of the target array was randomly selected from the remaining three colors with equal probability (33% of trials for each color), with the additional stipulation that the target and distractor were always different colors on a given trial. By comparing response time (RT) and accuracy on these *regularity-present blocks*, we could determine whether participants report the target orientation more quickly and accurately when the distractor appeared in a high-probability color.

After the first 12 blocks, where color regularities were present, participants performed eight more blocks of the discrimination task. During these last eight blocks, the target array color was chosen as before (33% of each nondistractor color). However, now the distractor array had an equal chance of being presented in any color (25% of trials for each color). Other than the change in color probabilities, the last eight blocks were identical to the first 12 blocks. Participants were not informed about a change in target/distractor color probabilities throughout the experiment. By comparing RT and accuracy in these *regularity-absent blocks*, we were able to determine if participants continue to suppress the distractor color even when this is no longer useful. Overall mean accuracy on the task was shown to the participants at the end of each block of the experiment (regularity-present and regularity-absent blocks).

Before starting the main session, participants completed a practice session of the task, which consisted of 60 trials of the orientation report task without any color regularities. There were 60 trials per block of the main session, and participants completed a total of 20 blocks over ~1 hr. Upon completing the experiment, we interviewed participants to determine whether they were aware of the color regularities. First, they were asked if they noticed any patterns or consistencies with the stimuli during the experiment. Second, they were told that the distractor array was usually one color and were instructed to guess the high-probability color.

#### Data analysis and statistical procedures

Trials with an RT 2.5 standard deviations above or below the individual participant’s mean RT, along with trials that were faster than 100 ms or slower than 2,500 ms, were removed from RT analyses. An average of 4% (*SD* = 1.51%) of trials were removed per participant after applying these exclusion criteria. We also excluded trials with an inaccurate orientation report from all RT analyses (13.8% of remaining trials). The task was intentionally made difficult to avoid ceiling effects, which explains the relatively high percentage of inaccurate trials. None of the experiments was preregistered.

We compared mean RT and accuracy on regularity-present blocks using paired-sample *t* tests to determine whether participants reported the target orientation more quickly and accurately when the distractor appeared in a high-probability color. To see if color suppression persisted when regularities were removed from the display, we computed a two-way repeated-measures analysis of variance (ANOVA), with color condition as the first factor (high-probability color vs. low-probability colors) and regularity presence as the second factor (regularity-present blocks vs. regularity-absent blocks). This analysis was followed by a *t* test comparison between mean RTs in the high- and low-probability distractor color conditions during regularity-absent blocks. Finally, we computed the linear correlation between suppression observed in regularity-present and regularity-absent blocks, where suppression was defined as the difference in mean RT between low-probability and high-probability distractor color trials. For all pairwise tests, we reported Bayes factor (BF) results using the bayesFactor package for MATLAB (Krekelberg, 2022). Evidence in favor of the null (BF_01_) is reported for nonsignificant tests, and evidence against the null is reported for significant tests (BF_10_). We used *d*_*z*_ as a metric of effect size for all *t*-test comparisons to account for shared variance in our repeated measures design (Lakens, 2013).

Seven subjects correctly identified the high-probability distractor color during a postexperiment interview, which did not differ from chance (binomial test: *p* = .393). All reported results were qualitatively the same when excluding participants who correctly reported the high-probability color. We analyzed the regularity-present suppression effect (low-probability − high-probability RT) separately for those who correctly identified the high-probability color and found no significant difference compared with those who were unaware of the color regularities (Supplemental Fig. 1a).

### Results and discussion

#### Regularity-present performance

**Fig. 2:**
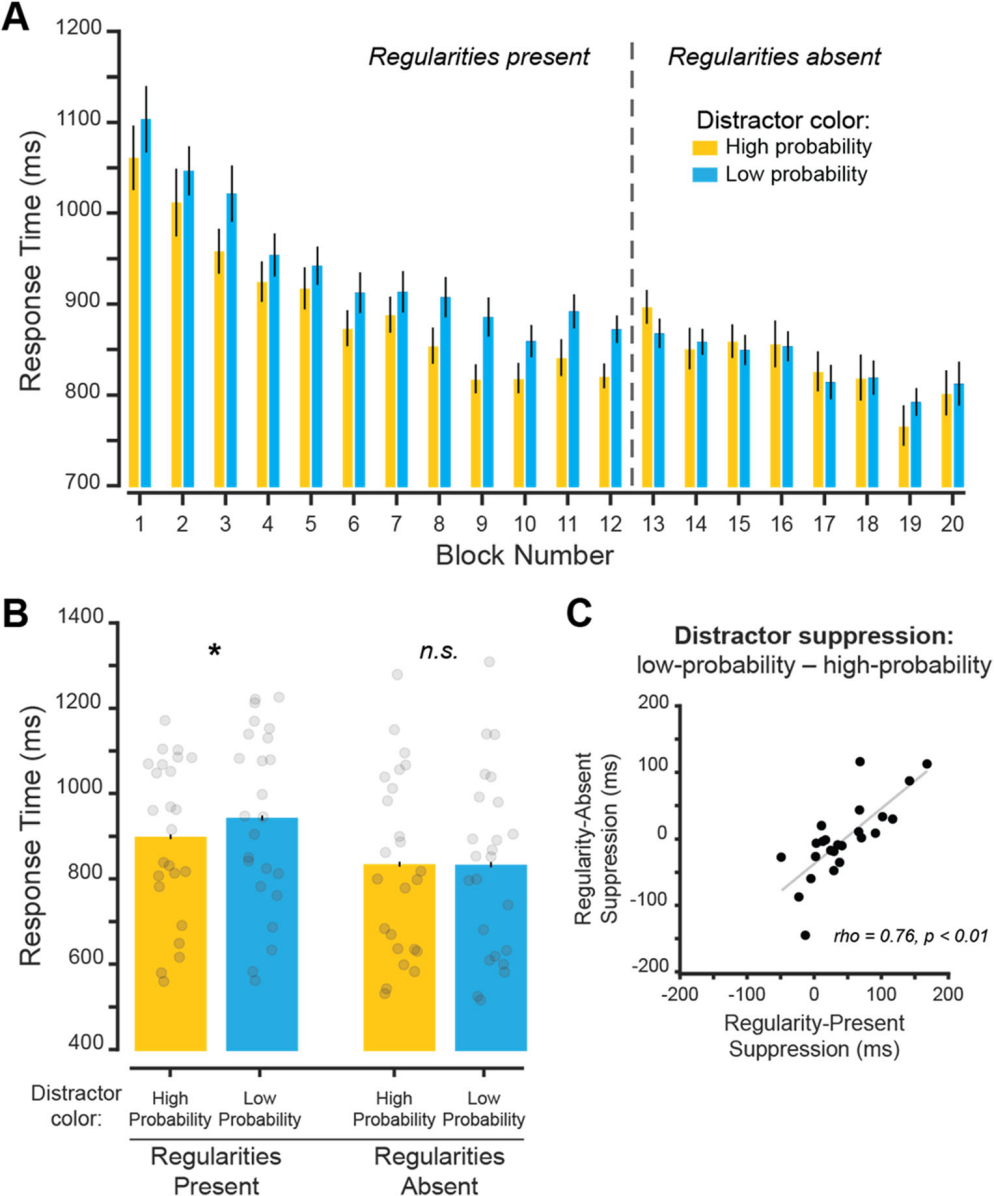
Experiment 1: High-probability distractor color is suppressed during learning and over an extended interval. **A** Mean RT for each block on trials with correct orientation reports. Dashed line indicates when distractor color regularities were removed from the display. **B** Mean RT across regularity-present and regularity-absent blocks for both high- and low-probability color conditions. Individual subject data points shown. Significant differences between color probability conditions indicated with * for *p* values < .05. **C** Correlation between suppression effect during regularity-present blocks and regularity-absent blocks. Suppression effects were computed as the difference in RT between the low- and high-probability color conditions. Error bars are within-subject standard error of the mean. (Color figure online)

First, we compared RT for target orientation discrimination across all task blocks throughout the experiment (Fig. 2A). Qualitatively, RTs were faster when the distractor appeared in the high-probability distractor color than when it appeared in another color. Additionally, RTs qualitatively sped up through the experiment. Next, we quantitatively established whether participants could more efficiently report the target orientation when a high-probability distractor color was present in the display during regularity-present blocks (Fig. 2B). We compared RTs (averaged across the initial regularity-present Blocks 1–12; Fig. 2A) on trials with the high-probability distractor and trials with another color distractor. Correct orientation reports on trials with high-probability distractor color were significantly faster than on trials with low-probability distractor colors, *t*(23) = 4.04, *p <* .001, *d*_*z*_ = 0.83, BF_10_ = 63.39. There was not a significant difference in orientation report accuracy between these trials (Table 1), *t*(23) = 1.55, *p* = .134, *d*_*z*_ = 0.32, BF_01_ = 1.63, indicating that the RT advantage is not due to a speed–accuracy trade-off. These results suggest that the high-probability distractor color was suppressed when stimulus regularities were present.

**Table 1:** Experiment 1 Accuracy (± *SEM*)

	Regularities present	Regularities absent
High-probability color	87.02% (1.08)	85.76% (1.10)
Low-probability color	83.65% (1.08)	84.06% (1.10)

#### Regularity-absent performance

Next, we identified whether distractor suppression persisted when color regularities were removed (Fig. 2B). To see if the difference between color conditions changed as a function of regularity presence, we performed a two-way repeated-measures ANOVA using RT data with distractor array color (high-probability color vs. low-probability colors) and regularity phase (regularity-present vs. regularity-absent) as factors. There was no effect of color condition, *F*(1, 23) = 4.01, *p* = .057, ${\eta}_p^2$ = 0.56, but there was a significant effect of phase *F*(1, 23) = 18.14, *p* < .001, ${\eta}_p^2$ = 0.96. The significant main effect of phase reflects the overall faster RTs during the later regularity-absent blocks as participants were getting better at the task. Importantly, there was a significant interaction, *F*(1, 23) = 32.64, *p* < .001, ${\eta}_p^2$ = 0.59. This result suggests that, at a group level, once regularities were removed from the display, the previously high-probability color was no longer suppressed. Follow-up comparisons between high- and low-probability distractor colors using data from the regularity-absent blocks are consistent with this conclusion: There was no significant difference between RTs when the distractor appeared in the previously high- versus low-probability distractor color, *t*(23) = 0.11, *p* = .917, *d*_*z*_ = 0.02, BF_01_ = 4.64.

While the above results suggest that, on average, participants no longer suppress the distractor color with learned regularities when the regularities are removed, we next considered the possibility that the magnitude of suppression during the regularity-present blocks within individual participants was carried over to the regularity-absent blocks. That is—do participants who most strongly suppress the learned distractor color when regularities are present also suppress the distractor color more than other participants when regularities are removed?

To test this, we calculated the correlation between the amount of suppression (defined as the difference in mean RT between low-probability and high-probability distractor color trials) during the regularity-present blocks and during the regularity-absent blocks (Fig. 2C). There was a strong positive relationship between these variables (*r* = .76, *p <* .001), indicating that subjects who suppressed the distractor during regularity-present blocks continued to suppress the distractor during regularity-absent blocks, despite no overall mean difference in the regularity-absent blocks across our participant sample (Fig. 2B).

Together, these findings show that a high-probability distractor color can be suppressed when regularities are present within a block. Critically, suppression occurred even when the arrays spatially overlapped, indicating that a specific color can be suppressed independent of spatial location. It is possible that suppression in this experiment was due to intertrial priming (Maljkovic & Nakayama, 1994), as suppression did not, on average, persist across our participant sample once regularities were removed. But an analysis of individual participants showed that those with stronger suppression effects when regularities were present continued to suppress the distractor color once regularities were removed (Fig. 2C). In addition to evaluating continued distractor suppression, previous studies have observed suppression effects when a target stimulus is presented at a learned distractor location (Britton & Anderson, 2020; Wang & Theeuwes, 2018). Since the target array was never presented in the high-probability color, it was not possible to conduct a similar analysis in Experiment 1. Experiment 2 was designed to better understand whether nonspatial color suppression mechanisms are transient or whether they result in suppression that persists over longer periods of time by including trials to directly measure suppression of the target array when regularities are removed (Fig. 1).

## Experiment 2

In Experiment 1, we showed that a regularly presented distractor color can be suppressed even when suppression cannot operate via a spatial location. Suppression occurred if regularities were present, but not once regularities were removed, consistent with a transient suppression effect such as intertrial priming (Maljkovic & Nakayama, 1994). However, spatial and color regularities have been shown to persist beyond the effects of priming in previous studies using visual search paradigms (Stilwell et al., 2019; Vatterott & Vecera, 2012). There was a hint of this effect at the level of individual subjects, where those who suppressed the distractor during regularity-present blocks continued to suppress the distractor during regularity-absent blocks. It may have been difficult to observe continued suppression during regularity-absent blocks due to relatively fast performance. In Experiment 2, we aimed to better probe the persistence of these suppression effects by evaluating performance in the regularity-absent blocks when the previously high-probability color appears as the target array (Britton & Anderson, 2020; Wang & Theeuwes, 2018). If the learned color is being suppressed, and the persistence of this effect was masked due to fast performance in the regularity-absent blocks, then suppressing the target array may allow for long-term suppression to be more readily observed via a *slowing* in discrimination performance when the target appears in the previously high-probability distractor color.

### Method

#### Participants

We recruited 24 new participants (18 female, mean age = 20 years) from the UCSB subject pool. Subjects were compensated with either course credit or $10/hr upon completing the task. None of the participants recruited for Experiment 2 participated in Experiment 1.

#### Design and procedure

Experiment 2 was identical to Experiment 1 during the regularity-present blocks (Blocks 1–12). The one critical change occurred in the regularity-absent blocks (Blocks 13–20; Fig. 1B). Similar to Experiment 1, the previously high-probability color had an equal chance of being the distractor array color (25% for each color). However, now the target array was presented in any of the four colors with equal probability (25% for each color), with the stipulation that the target and distractor colors were nonidentical. If suppression is due to long-term learning, then we would expect to see slower RTs when the target array was presented in the previously high-probability color. Furthermore, as in Experiment 1, it is possible that continued suppression effects persist in the regularity-absent blocks when the distractor array is presented in the high-probability color. However, due to the results of Experiment 1 (Fig. 2), we primarily expected this suppression effect to occur on the individual-subject level.

#### Analysis and statistical procedures

We removed trials that were faster than 100 ms and slower than 2,500 ms as well as trials 2.5 standard deviations above or below individual subject means. An average of 4.28% (*SD* = 1.59%) of trials were removed per participant. Trials with inaccurate responses were also removed from RT analyses (14.72% of trials). Six participants correctly reported their high-probability color, which did not differ from chance (binomial test: *p* = 0.578). Results are qualitatively the same when we exclude participants who correctly identified the high-probability color. Specifically, there was no difference in regularity-present distractor suppression between those who were aware and unaware of the high-probability color (Supplemental Fig 1B).

The same statistical tests computed for Experiment 1 were conducted in Experiment 2 when evaluating the influence of distractor array probabilities on performance. Additionally, to see if the high-probability color was suppressed during regularity-absent blocks, we computed a two-way repeated-measures ANOVA, with *target* array color as the first factor (previously high-probability distractor color vs. previously low-probability distractor color) and block as the second factor (regularity-absent Blocks 1–8). This was followed by a paired-samples *t* test comparing the mean RT across regularity-absent blocks of high- and low-probability target color conditions. Finally, we computed the linear correlation between *distractor* suppression in the regularity-present blocks and *target* suppression in the regularity-absent blocks to evaluate individual subject long-term suppression.

### Results and discussion

#### Regularity-present performance

**Fig. 3:**
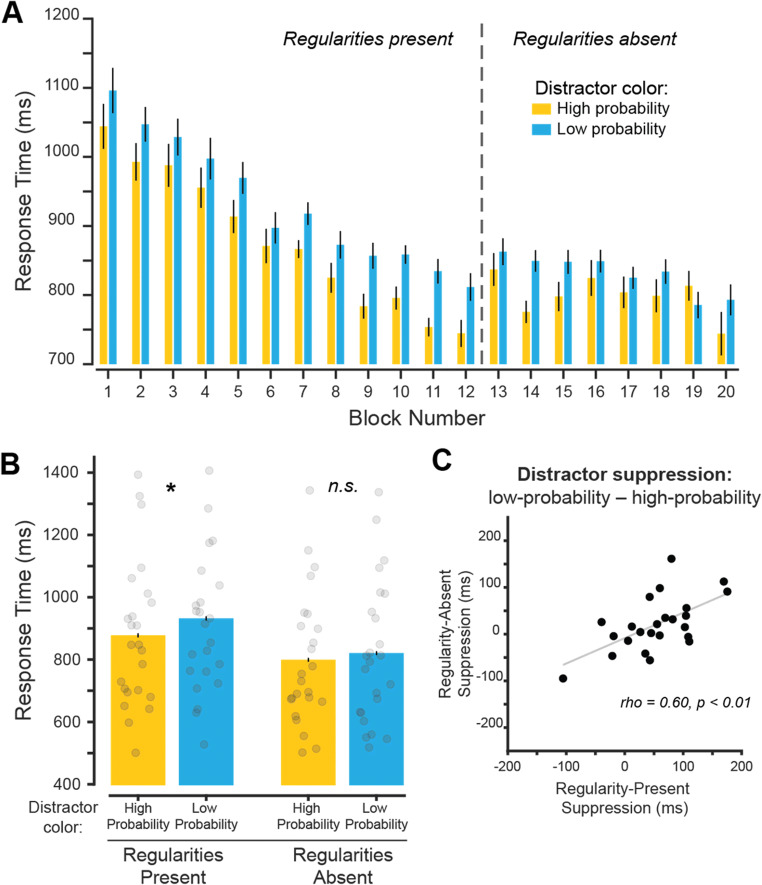
Experiment 2: High-probability distractor color is suppressed during learning and over an extended interval. **A** Mean RT for each block on trials with correct orientation reports. Dashed line indicates when distractor color regularities were removed from the display. **B** Mean RT across regularity-present and regularity-absent blocks for both high- and low-probability color conditions. Individual subject data points shown. Significant differences between color probability conditions indicated with * for *p* values < .05. **C** Correlation between suppression effect during regularity-present blocks and regularity-absent blocks. Suppression effects were computed as the difference in RT between the low- and high-probability distractor color conditions. Error bars are within-subject standard error of the mean. (Color figure online)

First, we verified that we could replicate the suppression effect observed in Experiment 1 during the regularity-present blocks (Fig. 2). Matching the results from Experiment 1, we saw that RTs were qualitatively faster when the high-probability color was shown as compared with the low-probability colors and that RTs increased throughout the experiment (Fig. 3A). We then compared the mean RTs from the regularity-present blocks between trials with a high-probability and low-probability distractor color (Fig. 3B). RT was faster in the high-probability color condition than the low-probability color condition, *t*(23) = 4.17, *p* < .001, *d*_*z*_ = 0.85, BF_10_ = 84.87. This replicates the main findings in Experiment 1, where the high-probability distractor color was suppressed when regularities were present, resulting in faster target discrimination performance. In addition, accuracy was greater for the high-probability distractor color condition (Table 2), *t*(23) = 2.77, *p* = .011, *d*_*z*_ = 0.57, BF_10_ = 4.53. Our accuracy results indicate that there was no speed–accuracy trade-off and that target identification accuracy was improved when the prevalent distractor color was present in the array.

**Table 2:** Experiment 2 Accuracy (± *SEM*)

	Regularities present	Regularities absent
High-probability color	86.94% (0.86)	85.45% (1.23)
Low-probability color	82.19% (0.86)	81.66% (1.23)

#### Regularity-absent performance

During the regularity-absent blocks, the previously high-probability color could be present in either the target or distractor array but was presented with the same probability as all other colors. Similar to Experiment 1, we conducted a two-way repeated-measures ANOVA, with *distractor color* as the first factor (high-probability color distractor vs. low-probability colors) and phase as the second factor (regularity-present vs. regularity-absent; Fig. 3B). There was a main effect of distractor color condition, *F*(1, 23) = 11.92, *p* = .002, ${\eta}_p^2$ = 0.69, and phase *F*(1, 23) = 21.08, *p* < .001, ${\eta}_p^2$ = 0.91. These findings demonstrate that participants were overall faster to respond when the distractor was shown in the high-probability color and that RTs were faster during regularity-absent blocks. Importantly, there was no interaction between these variables, *F*(1, 23) = 3.74, *p* = .066, ${\eta}_p^2$ = 0.14, which leaves open the possibility that suppression of the previously high-probability color continued when regularities were removed. However, follow-up comparison showed that distractor suppression across subjects did not persist into regularity-absent blocks, *t*(23) = 1.80, *p* = .086, *d*_*z*_ = 0.37, BF_01_ = 1.17.

To test if individual subjects continued to suppress the learned distractor color, we computed the correlation between the distractor suppression effect during the regularity-present blocks and the distractor suppression effect during the regularity-absent blocks (Fig. 3C). There was a positive correlation when comparing the regularity-present distractor suppression and regularity-absent distractor suppression (*r* = .60, *p* = .002). Consistent with Experiment 1, this result shows that participants who suppressed the high-probability color when regularities were present continue to suppress the color when regularities were removed.

**Fig. 4:**
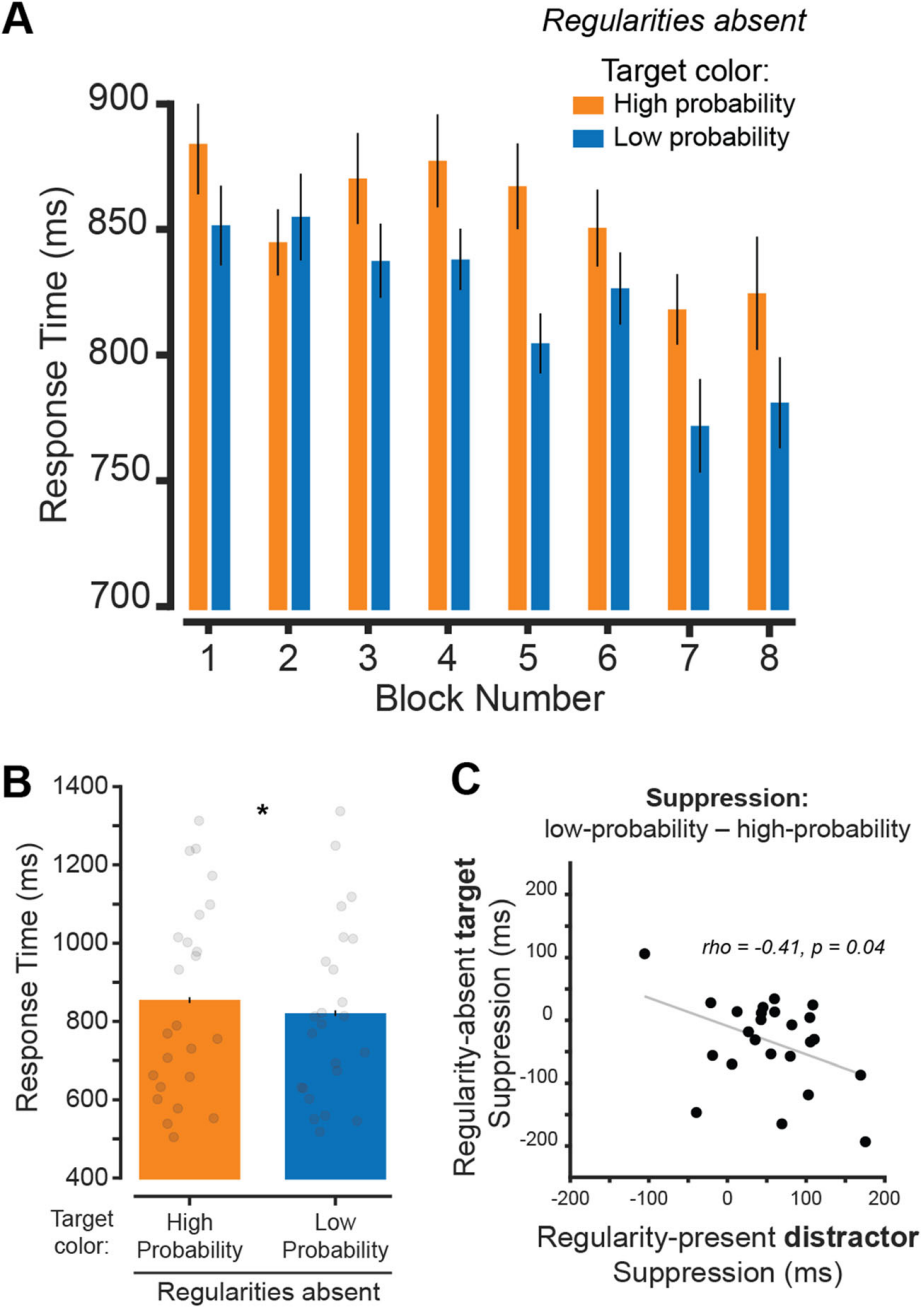
Learned distractor color is suppressed when used as target color after regularities are removed. **A** Mean RT for each regularity-absent block on trials where the target array was presented in the high- and low-probability color(s). **B** Mean RT across regularity-absent blocks for both the high- and low-probability color conditions. Individual subject data points shown. Significant differences between color probability conditions indicated with * for *p* values < .05. **C** Correlation between suppression effect during regularity-present blocks and regularity-absent blocks. Suppression effect for regularity-present blocks was computed as the difference in RT between the high- and low-probability *distractor* color conditions. Suppression effect for regularity-absent blocks was computed using high- and low-probability *target* color conditions. Error bars are within-subject standard error of the mean. (Color figure online)

Next, we compared RT across blocks when the *target array* was presented using either the previously learned high- or low-probability distractor color(s) to determine if suppression effects persist when regularities were no longer present (Fig. 4A). A two-way repeated measures ANOVA showed that there was a main effect of condition, *F*(1, 161) = 5.62, *p* = .027, ${\eta}_p^2$ = 0.16, as well as a main effect of block *F*(7, 161) = 2.82, *p* = .008, ${\eta}_p^2$ = 0.29. There was no interaction, *F*(7, 161) = 1.52, *p* = .165, ${\eta}_p^2$ = 0.06. A paired-sample *t* test showed a significant difference between target color conditions, *t*(23) = 2.37, *p* = .027, *d*_*z*_ = 0.48, BF_10_ = 2.16. Thus, when suppression was measured by presenting the *target array* in the learned high-probability distractor color, we observed persistent suppression after regularities were removed.

We then determined whether suppression effects during the regularity-present blocks in individual subjects predicted target suppression in regularity-absent blocks (Fig. 4C). There was a negative correlation between distractor suppression on regularity-present blocks (measured as the difference in low- vs. high-probability distractor color RTs) and target array suppression on regularity-absent blocks (measured as the difference in RT when the *target* was the previously low- vs. high-probability distractor color; *r* = −.41, *p* = .045), indicating that participants who suppressed the distractor when regularities were present (resulting in a faster target discrimination response) tended to respond slower to the target array when it was presented in the high-probability color. The negative correlation is expected since continued suppression of previously high-probability color should lead to worse performance when that color was present in the target array, even though the same suppression was helpful during the regularity-present blocks.

Overall, results from Experiment 2 showed that color suppression can occur independent of attenuation of specific spatial locations, replicating our main finding from Experiment 1. Additionally, suppression persisted even when stimulus regularities were no longer present, such that responses were slower when the target array was presented in the suppressed high-probability distractor color than when the target array was presented in a low-probability distractor color.

#### Intertrial priming: Analysis of aggregate data across experiments

As a final test of whether the nonspatial color suppression we observed was due to statistical learning (regularities learned throughout the experiment) or to intertrial priming (transient influence of previous trials), we computed the mean RT of each condition using only “switch” trials, or trials where the distractor probability was different from the distractor probability on the previous trial. We compared switch trials to “repeat” trials, where the distractor probability was the same as the distractor probability from the previous trial. This analysis allowed us to assess the individual contribution of priming, which is expected to result in a stronger effect on repeat than switch trials, and perseverant learning, which should still be present in switch trials. In addition to analyzing data after sorting each trial (*n*) based on the switch/repeat status of the previous trial (*n* − 1), we also looked at trials farther back in the experiment where the distractor probability matched/mismatched the current trial distractor probability in a serial manner (*n* − k). We sorted each trial (*n*) based on the trial label 1–8 trials previous (k = 1:8), because previous research has shown that priming no longer impacts RT after approximately seven trials (Maljkovic & Nakayama, 1994). Since this removes a large proportion of trials, and because Blocks 1 through 12 were identical in both experiments, we collapsed across data from both experiments to ensure adequate power (total *n* = 48).

**Fig. 5:**
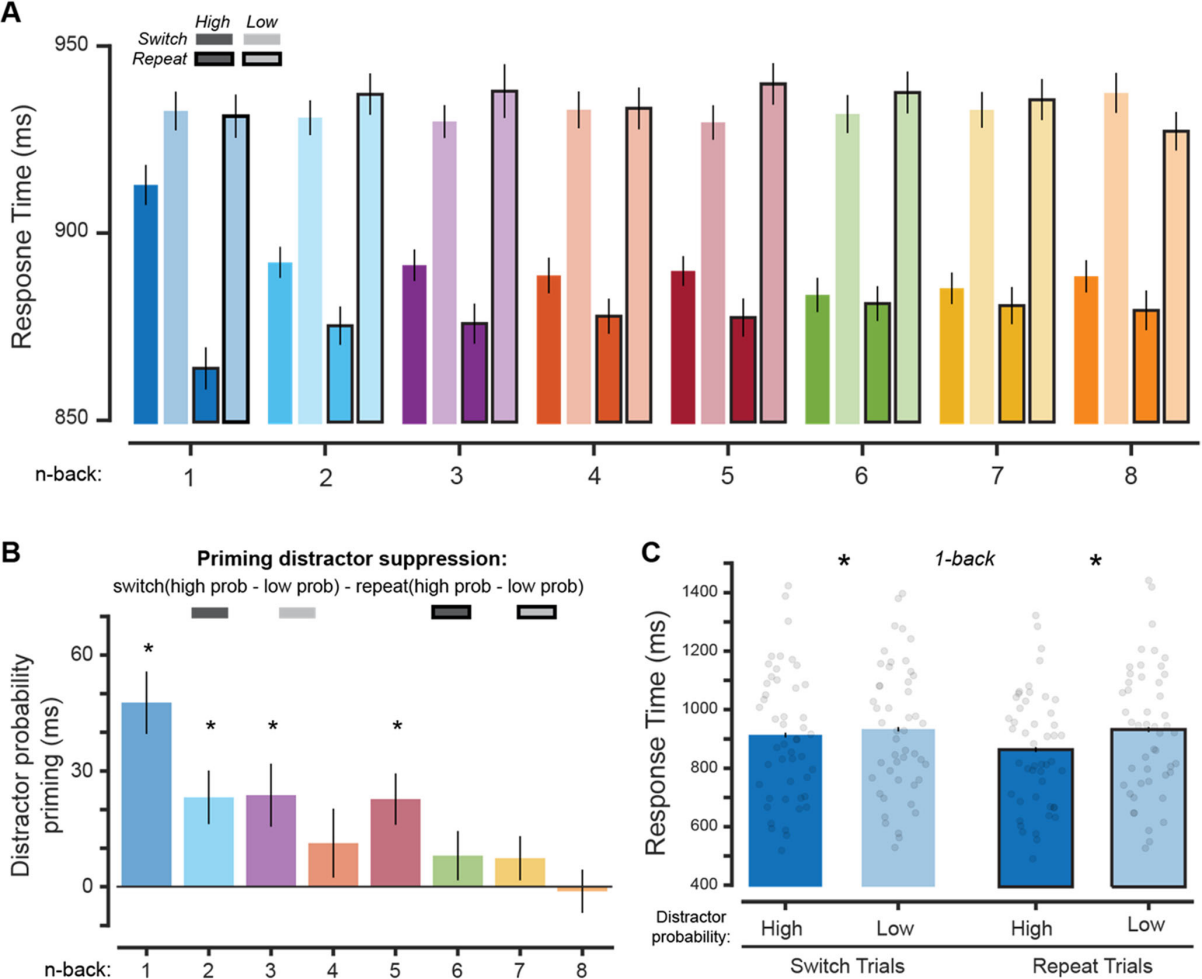
Suppression is not entirely explained by intertrial priming. **A** Mean RT was computed for the distractor array high-probability and low-probability conditions on trials where the previous trial used a different, or the same, distractor probability than that presented on the analyzed trial (“switch” and “repeat” trials, respectively). We performed this analysis serially, sorting by switch/repeat based on Trials 1–8 prior to the current trial. **B** Difference between “switch” and “repeat” trial suppression effects. Suppression was computed independently for switch/repeat trials as the difference between high- and low-probability distractor color conditions. Then, the difference between switch and repeat suppression effects were plotted, where positive values indicate greater suppression on repeat trials. * indicates significant difference, *p* < .05, one-sample *t* test. **C** Mean RT for the distractor array high- and low-probability color conditions for “switch” and “repeat” trials on *n* − 1 trials. * indicates significant difference, *p* < .05, paired *t* test. Data from both experiments were used to ensure enough power to detect an effect. Error bars are within-subject standard error of the mean. (Color figure online)

Figure 5A shows a significant three-way interaction between distractor probability (high- and low-probability), priming (switch and repeat), and serial position (*n* − 1 through *n* − 8), *F*(7, 329) = 3.88, *p <* .001, ${\eta}_p^2$ = 0.08. This demonstrates that distractor suppression was modulated by priming, but that this effect changed as a function of how far back in the trial sequence a repeat occurred. To better visualize the influence of priming at each serial position, we computed a priming distractor suppression value by first finding the difference in RT between the high- and low-probability distractor color conditions independently for switch and repeat trials, then computing the difference between these values. Positive values of this measure indicate greater distractor suppression on repeat trials (Fig. 5B).^1^

Priming indeed had a diminishing effect on RT the farther back a repeat occurred in the trial sequence, with most influence absent after *n* − 5. Importantly, when comparing the high- and low-probability distractor color conditions using only *n* − 1 switch trials (where intertrial priming had the strongest influence; Fig 5B), RTs were still significantly faster when the high-probability color was shown, *t*(47) = 2.25, *p* = .029, *d*_*z*_ = 0.32, BF_10_ = 1.54 (Fig. 5C). This is additional evidence suggesting that feature suppression is due, at least in part, to long-term learning of stimulus regularities.

## General discussion

The current study was designed to understand whether a distracting stimulus defined based on its color could be suppressed independent of spatial location. If true, target discrimination performance should be improved when a high-probability distractor color is present at the same location of a target stimulus as compared with when any low-probability distractor color is presented. We tested this by showing participants two overlapping line arrays, where they had to report the orientation of the array with the most lines (Fig. 1A). During regularity-present blocks, the distractor array was usually presented in one color. Over the course of both experiments, RTs were faster when this high-probability color was present in the distractor array relative to one of the other low-probability colors, indicating that the distractor color was suppressed (Figs. 2B and 3B). Distractor suppression persisted when color regularities were removed from the display for subjects utilizing them during regularity-present blocks, indicating that suppression cannot be fully explained by priming (Figs. 2C and 3C). In Experiment 2, we found stronger evidence in favor of long-term suppression: RTs were slower when the target array was presented in the high-probability color (Fig. 4), and this suppression persists when we only analyzed switch trials during regularity-present blocks (Fig. 5).

Our findings build on the growing literature demonstrating feature suppression through repeated exposure to regularly presented visual search singletons (Failing et al., 2019; Gaspelin & Luck, 2018; Stilwell & Gaspelin, 2021; Stilwell et al., 2019; Vatterott & Vecera, 2012). Importantly, the effects in the present study were identified when spatial suppression mechanisms could not be used to lower the prioritization of distracting items. In all of the aforementioned studies, visual search tasks were employed, which have been useful in identifying when particular display statistics are used to guide search behavior (Stilwell et al., 2019) as well as how regularities may interact within and between feature dimensions to modulate suppression (Failing et al., 2019). However, to further understand how these regularities are deployed, it is important to understand each one in isolation. Our stimulus, in which *only* color regularities could contribute to guiding suppression, could be a useful tool for future studies to isolate feature-specific suppression mechanisms from their spatial counterparts.

Potentially contrasting with findings of feature-specific suppression are studies indicating that only stimulus *locations* can be deprioritized (Moher & Egeth, 2012; Theeuwes, 2010). For example, Moher and Egeth (2012) had participants perform a target detection task where a cue was given at the start of each trial. This cue was informative about the color of distractors in an upcoming multi-item display, where each item occupied a unique location. Target detection was faster when an informative distractor cue was provided as compared with a neutral cue, but this effect was only observed when the distractor location was attended prior to the onset of a target. This result led to their *search-and-destroy* hypothesis, which states that a location needs to be selected first and then a distractor presented in a learned feature can be suppressed. Further evidence suggesting that suppression is location-dependent comes from their Experiment 4, as suppression was not improved when there were several distractors of the same color, inconsistent with accounts of feature-based attention in which a specific feature value can be up/down-regulated across the entire screen simultaneously (Maunsell & Treue, 2006; Treue & Trujillo, 1999).

There are two noteworthy differences between our study and Moher and Egeth (2012). First, as mentioned previously, the spatially overlapping stimulus used in the current study discouraged the use of any spatial suppression mechanisms, as it would not have benefited target detection. Second, Moher and Egeth (2012) used cues to direct volitional control towards suppressing task-irrelevant information. In our study, subjects were unaware of the display statistics, yet their performance was modulated by the presence of a high-probability color. It appears that top-down control cannot be used to suppress distracting information in a parallel feature-based manner, but implicit mechanisms allow for a more global suppression. While this may be the case, a potential downside to implicit learning is that suppression persists even when it is no longer useful, as was evident in our Experiment 2 (Fig. 4B–C), whereas top-control can be implemented on a trial-by-trial basis (Cunningham & Egeth, 2016). Overall, our results provide strong evidence that feature-specific suppression obtained through statistical learning can occur *independent* of top-down spatial suppression operations such as reactive control (Theeuwes, 2010) or search-and-destroy processes (Moher & Egeth, 2012).

While we ruled out reactive spatial mechanisms as a possible alternative to learned feature suppression, the current findings are unable to address whether suppression exclusively occurred proactively or reactively. It could be the case that the distractor array is less likely to be selected when presented in the high-probability color (Gaspelin et al., 2019), or the distractor array is still selected but the high-probability color is rapidly suppressed through reactive feature suppression. Without explicit knowledge of the color regularities, both line arrays need to be attended to determine which is the target. Thus, we speculate that reactive mechanisms were deployed when attending our stimuli. However, it is plausible, especially during regularity-present blocks, that a proactive mechanism was also used as participants implicitly learned the high-probability color. Ultimately, both strategies can be implemented (Geng, 2014). For instance, it is often more efficient to proactively ignore distracting stimuli but, since these regularities may not persist—as is the case in our regularity-absent blocks—it can be beneficial to allow for learned distractors to occasionally capture attention to update learned regularities. This is even more effective with a reactive mechanism to quickly disengage from stimuli as long as they are still distracting.

How do our results fit with priority map theory? Within this framework, maps corresponding to individual feature dimensions are integrated into a feature-agnostic priority map (Itti & Koch, 2001; Wolfe, 1994). Locations with the greatest prioritization are selected for the allocation of attention. Mechanisms for distractor suppression generally fit nicely within this model, as they explain how locations within these maps are deprioritized (Failing et al., 2019; Luck et al., 2020). Whenever feature-specific suppression is engaged, modulations are thought to occur within the corresponding feature map. For example, a regularly presented red singleton will have lower activation in the red feature map, which results in lower activation in the summed priority map. It is difficult to reconcile our results exactly within this structure, as suppressing the distractor location would also deprioritize the target item due to their shared location. Rather than specific locations being the target of prioritization, others have proposed that modulation can occur at the level of individual objects (Shomstein, 2012; Shomstein & Yantis, 2002), even when they are occluded (Moore et al., 1998). According to this account, after directing spatial attention, goal-relevant objects at that location are selected before other less-relevant objects. This mechanism of object-based attention is compatible with the spatially overlapping stimuli used in the current study. When considered within the context of feature maps, in addition to the high-probability color being suppressed, the orientation of the lines associated with the high-probability color would be suppressed allowing for the other object in the display to be selected first. Future work can manipulate the statistics in this paradigm to tease apart when objects, features, and/or locations are suppressed.

Rather than a suppressive reweighting of objects within the priority map framework, it could be the case that distractor statistics are used to shift or enhance the representation of the three possible target colors. Recent evidence shows that when distractors are regularly presented in colors that are linearly separable from the target color in feature space, the representation of a target color shifts away from the color of distractors (Navalpakkam & Itti, 2007; Witkowski & Geng, 2019; Yu & Geng, 2019). By shifting the target representation, it makes it harder for distractors with a similar color as the target to capture attention (Duncan & Humphreys, 1989). In the current study, it is possible that the representation of each target color was shifted away from the learned distractor color to improve performance. However, there are a couple of aspects of our design that are difficult to reconcile with this account. First, the high-probability color was only the most likely distractor color—the other three colors were the distractor on some trials. Yu & Geng (2019) showed that when distractor colors were sampled from either side of feature space, the target color representation no longer shifted. Second, these previous studies have primarily investigated how distractor statistics influence target representations, but there has yet to be a study showing how implicitly learned distractor information is modulated in similar visual search paradigms, so it is unclear whether both targets and distractors are influenced. Regardless, this remains an interesting mechanism and further studies should investigate the degree of influence distractor statistics have on both targets and distractors.

In both experiments, there was evidence that learned distractor suppression may be a variable characteristic across individuals since suppression effects persisted in regularity-absent blocks for participants who showed an effect during regularity-present blocks (Figs. 2C, 3C and 4C). This may not come as a surprise, as similar findings are apparent in the working memory literature (Luria et al., 2016). As an example, individuals who perform well on memory tasks tend to be better at ignoring distracting information (Vogel et al., 2005). While speculative, it is possible that the ability to leverage distractor statistics to prioritize target information is related to the ability to prevent distracting information from entering visual working memory. In fact, the distractors used in the primary experiment of Vogel et al. (2005) were always red—a feature regularity that could be used in a manner consistent with learned suppression. This is further supported by the strong relationship between visual working memory and attention (Awh & Jonides, 2001; Bahle et al., 2018; Olivers et al., 2006). However, additional studies are needed to directly test whether distractor suppression observed in studies of selection history is related to the ability to prevent irrelevant information from entering working memory.

### Conclusion

It is imperative to suppress distracting information for effective selection of relevant stimuli in service of goal-oriented behavior. Mounting evidence has shown that locations corresponding to a distractor can be suppressed (Gaspelin et al., 2015; Stilwell et al., 2019; Wang & Theeuwes, 2018), but it is important to understand whether nonspatial features can be inhibited when space-based suppression is not beneficial. Our study showed that when overlapping stimuli are presented, a high-probability distracting color is suppressed to improve target discrimination performance. This suppression persisted even when regularities were removed from the display, indicating that learned statistics contributed to this effect. Overall, we provide strong evidence that features can be suppressed independent of spatial location.

## Supplementary information

ESM 1 (DOCX 66 kb)

## Footnotes

1 Our analysis is unable to completely exclude priming as a contributing factor, as we did not account for repeat trials between or after the analyzed trials (e.g., Trials 1 through 7 when analyzing *n* − 8). This is because trials with no repeats between/after trial *n* were exceedingly rare, making it difficult to interpret results. However, our findings do clearly demonstrate that priming cannot entirely account for learned color suppression.
