# Supplementary material for: Learned feature regularities enable suppression of spatially overlapping stimuli
Source: Atten Percept Psychophys. 2022 Nov 23;85(3):769–84. doi: 10.3758/s13414-022-02612-1 (PMC10066085; doi:10.3758/s13414-022-02612-1)
Supplement: Supplementary file 1 — (DOCX 66 kb) [file 13414_2022_2612_MOESM1_ESM.docx]

| 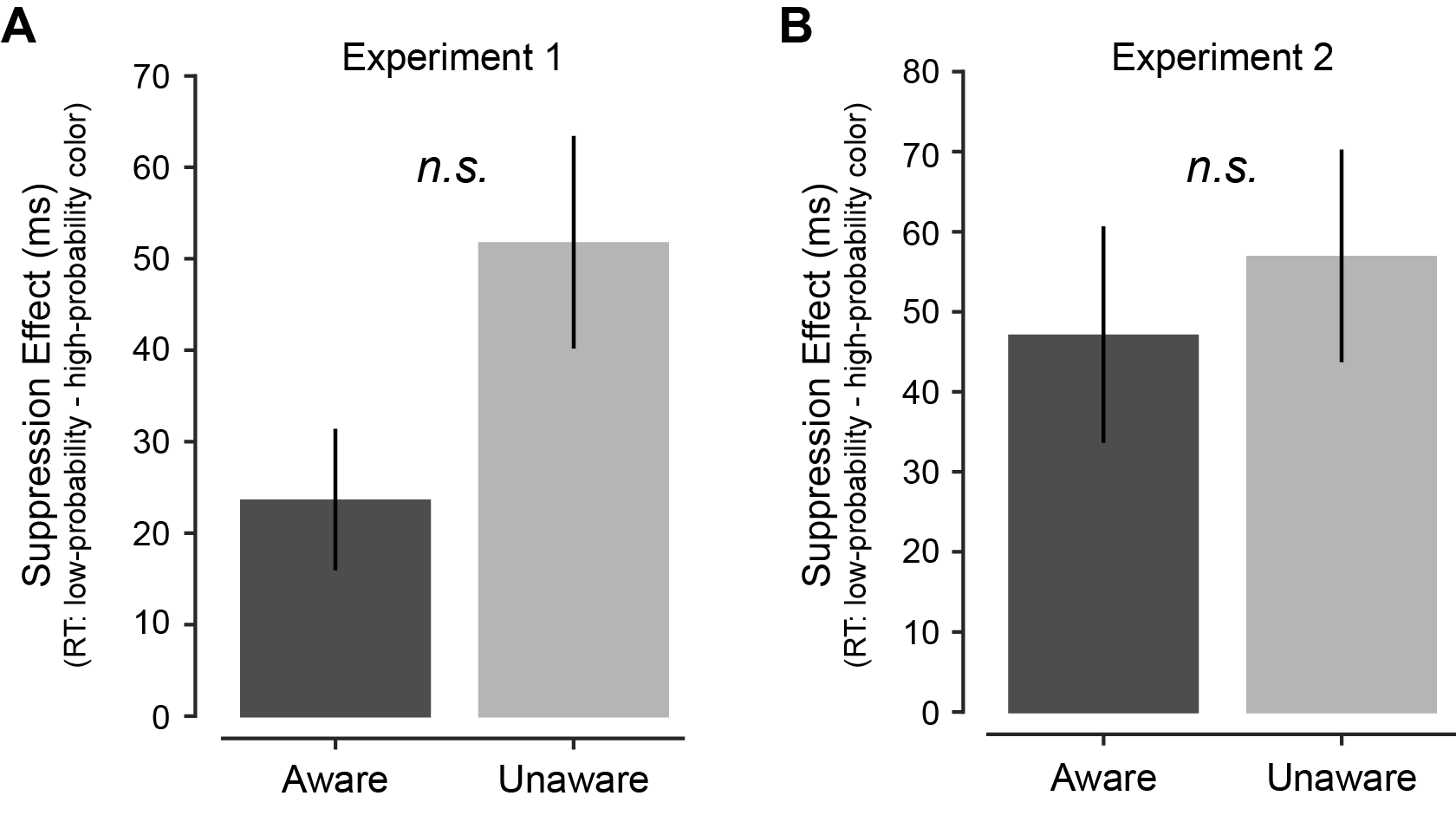 |
| --- |
| **Supplemental Figure 1: Distractor suppression does not require awareness.** Suppression effect (target discrimination RT for low-probability minus high-probability distractor color; regularity-present trials) compared across groups of participants aware or unaware of distractor color regularities. For each Experiment (A: Expt 1; B: Expt 2), we split participants into groups based on whether they correctly reported the regular distractor color (“Aware”) or not (“Unaware”). Distractor suppression did not differ between these groups, and qualitatively, distractor suppression was stronger when participants did not correctly detect regularities. Error bars are between-participant SEM. |
